# Proliferation-Linked Apoptosis of Adoptively Transferred T Cells after IL-15 Administration in Macaques

**DOI:** 10.1371/journal.pone.0056268

**Published:** 2013-02-13

**Authors:** Carolina Berger, Michael Berger, Brian C. Beard, Hans-Peter Kiem, Theodore A. Gooley, Stanley R. Riddell

**Affiliations:** 1 Clinical Research Division, Fred Hutchinson Cancer Research Center, Seattle, Washington, United States of America; 2 Department of Medicine, University of Washington School of Medicine, Seattle, Washington, United States of America; 3 Focus Group “Clinical Cell Processing and Purification”, Institute for Advanced Study, Technical University of Munich, Munich, Germany; Saint Louis University School of Medicine, United States of America

## Abstract

The adoptive transfer of antigen-specific effector T cells is being used to treat human infections and malignancy. T cell persistence is a prerequisite for therapeutic efficacy, but reliably establishing a high-level and durable T cell response by transferring cultured CD8^+^ T cells remains challenging. Thus, strategies that promote a transferred high-level T cell response may improve the efficacy of T cell therapy. Lymphodepletion enhances persistence of transferred T cells in mice in part by reducing competition for IL-15, a common γ-chain cytokine that promotes T cell memory, but lymphodepleting regimens have toxicity. IL-15 can be safely administered and has minimal effects on CD4^+^ regulatory T cells at low doses, making it an attractive adjunct in adoptive T cell therapy. Here, we show in lymphoreplete *macaca nemestrina*, that proliferation of adoptively transferred central memory-derived CD8^+^ effector T (T_CM/E_) cells is enhanced in vivo by administering IL-15. T_CM/E_ cells migrated to memory niches, persisted, and acquired both central memory and effector memory phenotypes regardless of the cytokine treatment. Unexpectedly, despite maintaining T cell proliferation, IL-15 did not augment the magnitude of the transferred T cell response in blood, bone marrow, or lymph nodes. T cells induced to proliferate by IL-15 displayed increased apoptosis demonstrating that enhanced cycling was balanced by cell death. These results suggest that homeostatic mechanisms that regulate T cell numbers may interfere with strategies to augment a high-level T cell response by adoptive transfer of CD8^+^ T_CM/E_ cells in lymphoreplete hosts.

## Introduction

The adoptive transfer of antigen-specific CD8^+^ effector T (T_E_) cells is being employed with increasing success in the treatment of human diseases [1]–[8]. Effective T cell therapy typically requires that transferred T cells persist in vivo for a considerable duration [9], [10]. However, the survival of ex-vivo expanded antigen-specific CD8^+^ T_E_ cells in most clinical trials has been unpredictable and often short, even when IL-2 is provided to promote T cell survival [10]–[12]. We have used a nonhuman primate (NHP) model to investigate strategies to improve T cell transfer, and previously identified a role for cell-intrinsic properties of memory CD8^+^ T cell subsets in determining persistence after adoptive transfer [13]. In this model, we found that the administration of cytomegalovirus (CMV)-specific CD8^+^ T_E_ clones derived from central memory (T_CM_) but not effector memory (T_EM_) cells to lymphoreplete animals resulted in the establishment of a durable functional reservoir of memory cells in vivo [13]. Subsequently, adoptive T cell transfer studies using human T cells have focused on inducing or retaining central memory properties during in vitro expansion of T_E_ cells, and demonstrated a correlation of T_CM_ properties with improved T cell persistence in vivo [8], [14]–[18].

A characteristic of the CMV-specific T_CM_-derived CD8^+^ T_E_ (T_CM/E_) clones observed in our studies in lymphoreplete macaques was that IL-15 maintained their survival in vitro [13]. By contrast, the CMV-specific CD8^+^ T_EM/E_ clones were much less responsive to the effects of IL-15 in vitro [13]. This motivated us to examine whether administering IL-15 with transferred CD8^+^ T_CM/E_ cells in vivo would enhance the magnitude of the transferred T cell response in vivo. IL-15 is a common γ-chain cytokine that is required to maintain survival of endogenous CD4^+^ and CD8^+^ memory T (T_M_) cells [19]–[24]. We previously showed in NHPs that administering IL-15 subcutaneously in doses of 2.5–10 µg/kg every 3 days was safe, and transiently increased proliferation of endogenous NK cells and CD8^+^ T cells, without a major increase in CD4^+^FoxP3^+^ T regulatory (T_reg_) cells [25]. Here, we examine the effects of the combined administration of IL-15 and gene-marked clonal or polyclonal CD8^+^ T_CM/E_ cells in lymphoreplete macaques on the persistence, migration, and phenotype of the adoptively transferred T cells.

## Materials and Methods

### Ethics Statement, Animals, and Cell Transfer

Adult *macaca nemestrina* were used in this study. The NHPs were housed at the Washington National Primate Research Center (WaNPRC) under American Association for Accreditation of Laboratory Animal Care approved conditions. The study was performed according to recommendations in the Guide for the Care and Use of Laboratory Animals of the National Institutes of Health. The Institutional Animal Care and Use Committee approved the experimental protocol (University of Washington #4159-01; Fred Hutchinson Cancer Research Center (FHCRC) #1638). The macaques were housed in pairs in run-through connected cages according to USDA standards. Food consisted of Lab Diet 5049 (high fiber) and food grade produce. Water was provided ad libitum via drinking valves in the cages. The Environmental Enhancement Plan and psychological Well-Being Program included, as required by federal law, diverse enrichment tools (perches, toys, puzzle feeders, food treats, foraging experiences, wall-mounted mirrors). The animals were observed at least twice daily by trained personnel of the WaNPRC staff. To minimize pain from the procedures, analgesics were administered for a sufficient time. All animals were returned healthy to the colony after the completion of the experiment. CMV-specific CD8^+^ T_CM/E_ clones or polyclonal CD8^+^ T_CM/E_ (5×10^8^/kg) were infused intravenously alone or with human recombinant IL-15 (provided by Amgen) [26], administered subcutaneously every 3 days for 9 doses at a dose of 10 µg/kg, except for macaque M07191 that received a dose of 5 µg/kg [25]. Complete blood counts and serum chemistry were measured in accredited laboratories. Persistence of transferred T_CM/E_ cells was measured by flow cytometry using macaque truncated CD19 (ΔCD19) or CD20 markers introduced by retroviral gene transfer, and by quantitative real-time PCR (qPCR) for unique vector sequences [13], [27].

### Retroviral Transduction and Expansion of CMV-specific CD8^+^ T_CM/E_ Clones or Polyclonal T_CM/E_ Cells

Isolation of CMV-specific CD8^+^ T_CM/E_ clones, gene marking, expansion, and specificity analysis of the CMV-specific CD8^+^ T_CM/E_ clones was performed as described [13], [27]. Polyclonal CD8^+^ T_CM/E_ cells were derived from sort-purified CD95^+^CD62L^+^CD8^+^ T cells. The majority of the CD8^+^ T_CM_ cells express both CD62L and CCR7, respectively, but there is evidence for some heterogeneity with regard to the CCR7 expression in the CD8^+^ T_CM_ subset [28]–[30]. To enable comparison with prior results in this model, we used CD62L rather than CCR7 as a sorting parameter to isolate T_CM_. Selecting on CD62L provided cell populations that were >92% CD62L^+^, of which 61–97% were CCR7^+^ (Fig. S1). Aliquots of the selected T cells were stimulated with anti-CD3 (BD Biosciences) and anti-CD28 monoclonal antibodies (mAbs), γ-irradiated human peripheral blood mononuclear cells (PBMC) that were obtained via leukapheresis from volunteer donors (FHCRC, IRB #868) and γ-irradiated human EBV-lymphoblastoid lymphocytes from a validated cell line obtained from donor TM (obtained from City of Hope, Duarte, CA) [31]–[33]. Human recombinant IL-2 (Chiron, Emeryville, CA) was added at intervals at a dose of 50 U/mL as previously described [32], [33]. On day 2 and 3, T cells were transduced with ΔCD19 or CD20 retroviruses, and ΔCD19^+^ or CD20^+^ T cells were then enriched by immunomagnetic selection (Miltenyi) and cryopreserved for expansion and infusion [13].

### Flow Cytometry

PBMC and T cells were stained with fluorochrome-conjugated mAbs to CD3 (SP34), CD4, CD8, CD16, CD20, CD28, CD62L, CCR7, CD95, CD122 (IL-2/15 Receptor (R) β-chain) (BD Biosciences), CD19 and CD127 (IL-7Rα, Beckman Coulter), and isotype-matched controls. For multi-parameter flow cytometry, T cell subsets were identified with the following markers: naïve T cells (T_N_): CD95^low^CCR7^+^; T_CM_ : CD95^+^CCR7^+^; and T_EM_ CD95^+^ CCR7^–^ [25], [27], [34]. For intracellular staining with Ki-67 or Granzyme B mAbs (BD Biosciences), cells were permeabilized using Cytofix/Cytoperm. Samples were stained for surface expression of Annexin V and intracellular Ki-67 expression according to the manufacturer’s instruction (eBioscience) [35]. In some experiments, a Vybrant FAM Poly Caspases assay kit was used according to the manufacturer’s instruction (Invitrogen). CD4^+^ T_reg_ cells were enumerated as described [25]. Samples were analyzed on a FACSCalibur or LSR-II instrument and using FlowJo software (Tree Star, Inc.).

### Cell Viability Assays and CFSE-labeling

Aliquots of T_CM/E_ cells were washed 14 days after CD3/CD28 stimulation and plated at 2×10^6^ cells/well in T cell media alone or with IL-15 (0.05−10 ng/mL, R&D Systems). Viability was assessed every 3−4 days by trypan-blue dye-exclusion. In some experiments, cells were labeled with CFSE or CellTrace (Invitrogen) as described [13], and viability of T cells that had divided was analyzed by flow cytometry after staining with anti-CD8 and anti-CD3 mAbs, Annexin V, and Propidium Iodide (PI) or a Vybrant FAM Poly Caspases assay kit.

### Telomere Length Analysis

The average length of telomere repeats in lymphocytes was measured by automated flow-FISH [36]. To convert the fluorescence measured in sample cells hybridized with the telomere PNA probe into kb of telomere repeats, fixed bovine thymocytes with known telomere length served as internal control [36].

### IL-15 Elisa

Plasma samples were analyzed for IL-15 using an ELISA kit according to the manufacturer’s instruction (R&D Systems). The detection level of the assay is 0.3−1.2 pg/mL.

### Retrovirus Integration Site (RIS) Analysis

RIS analysis was performed using modified genome sequencing (MGS)-PCR [37]. To amplify vector-genome junctions, dsDNA was amplified in sequential, nested exponential PCR [37]. Multiplex identifier (MID1-100, Roche) tags were incorporated in the nested LTR-specific primers that act as 10 bp barcodes. RIS were then examined using a massively paralleled pyrosequencing method. Briefly, RIS were gel-purified to isolate DNA fragments (∼600–1000 bp) and sequenced on the 454-titanium platform (R. J. Carver Biotechnology Center, University of Illinois). RIS were mapped relative to genomic elements as described [38].

### Statistical Analysis

Statistical analysis of changes in absolute numbers of endogenous cell populations during the IL-15 treatment was performed as described [25]. The difference from baseline for week 1, 2, and 3 was calculated for each animal. We determined the average of these 3 values for each animal and used a one-sample t-test to examine whether these values were different from zero. The area under the curve (AUC) of the T cell persistence was used to compare the persistence-level. Two-tailed paired t-test was used for comparison between matched paired groups. Statistical analysis was performed using Graphpad Prism Software.

## Results

### Safety and Immunomodulatory Effects of Administering IL-15 with Transferred CMV-specific CD8^+^ T_CM/E_ Clones

**Figure 1:**
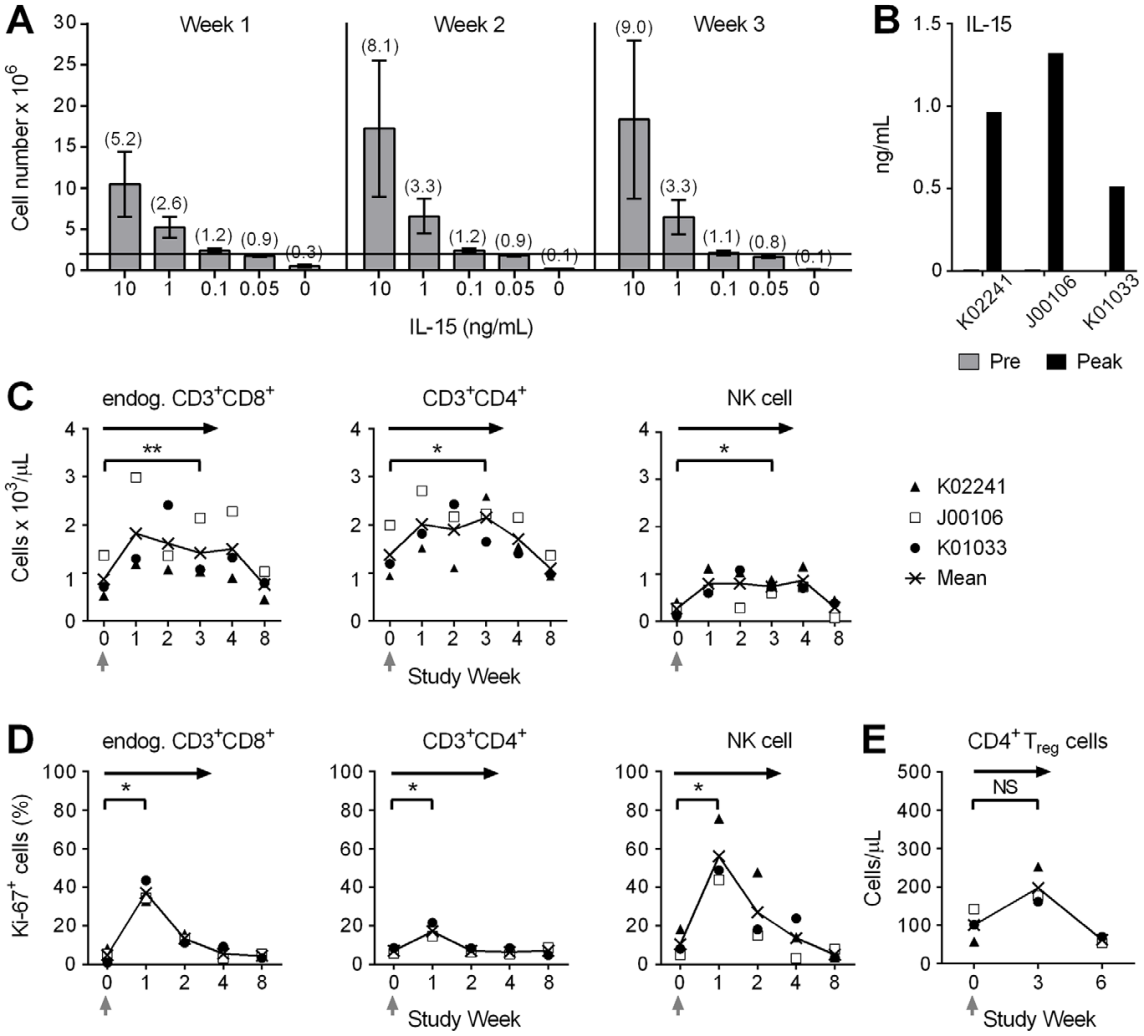
Effects of IL-15 on CD8^+^ T_CM/E_ in vitro and endogenous cells in vivo. (A) In vitro survival of CMV-specific CD8^+^ T_CM/E_ clones. T_CM/E_ cells were stimulated for 14 days with anti-CD3/CD28 mAbs, γ-irradiated feeder cells, and IL-2 (50 U/mL). Aliquots were washed and plated in media alone, or media containing 0.05−10 ng/mL of IL-15. Cell numbers of 6 T_CM/E_ clones from different macaques were determined by counting viable cells at the indicated time points. Shown are mean ± SEM, and the fold-increase compared to day 0 in parenthesis. (B) IL-15 levels. Samples of plasma obtained from macaque K02241, J00106, and K01033 prior to treatment (gray bars) and 2 hours after the third dose of IL-15 given on day 6 after the T cell infusion (black bars), were examined for IL-15. (C) Absolute numbers of endogenous ΔCD19^–^CD3^+^CD8^+^ and CD3^+^CD4^+^ T cells, and CD16^+^ NK cells/µL of peripheral blood of macaque K02241 (closed triangle), J00106 (open square), and K01033 (closed circle). PBMC were obtained before, during, and after the CD8^+^ T_CM/E_ infusion with IL-15 and stained with mAbs to CD3, CD4, CD8, and CD16. (D) Intracellular Ki-67 expression. The percent (%) of Ki-67^+^ cells in endogenous ΔCD19^–^CD3^+^CD8^+^ or CD3^+^CD4^+^ T cells, and CD16^+^ NK cells is shown for each of the 3 macaques. (E) Absolute numbers of CD4^+^ T_reg_ cells/µL in peripheral blood. PBMC were obtained at the indicated times from the macaques, stained with mAbs to CD3, CD4, CD25, and FoxP3, and examined by flow cytometry after gating on CD3^+^CD4^+^ T cells. (C–E) The black line connects the mean (×) at each time point. The black arrows indicate the duration of the IL-15 treatment and the gray arrows indicate the T_CM/E_ infusion. NS, not significant; * *P*<0.05; ** *P*<0.01.

Prior to the in vivo experiments, we evaluated the effect of IL-15 on the in vitro survival of CMV-specific CD8^+^ T_CM/E_ clones from six animals cultured in the absence of T cell receptor ligation. We found that ≥0.05 ng/mL of IL-15 enhanced survival of CMV-specific CD8^+^ T_CM/E_ clones compared with media alone, and that ≥1 ng/mL of IL-15 increased cell numbers over a 3-week culture period (Fig. 1A).

CMV-specific CD8^+^ T_CM/E_ clones from three animals (K02241, J00106, and K01033) were gene-marked with retroviral vectors encoding for ΔCD19 or CD20. There was no apparent difference in the T_CM/E_ clones from the animals in growth, telomere length, or cytotoxic activity (Fig. S2A–C). We adoptively transferred the ΔCD19-marked CMV-specific CD8^+^ T_CM/E_ clone (5×10^8^ cells/kg) to each macaque and administered IL-15 (10 µg/kg) subcutaneously every 3 days. There were no clinical toxicities apart from a transient rash and mild pruritus in animal K01033. Pretreatment IL-15 levels were <0.02 ng/mL and the peak-level of IL-15 on day 6 ranged from 0.51−1.32 ng/mL (Fig. 1B), consistent with results of our prior study of IL-15 alone, and ten-fold above that required to support T cell survival in vitro [25]. There were no major alterations in neutrophil counts, and the absolute numbers of CD8^+^ and CD4^+^ T cells and NK cells showed a statistically significant increase during the 3 weeks of IL-15 treatment (Fig. 1C). These changes were accompanied by a statistically significant increase in the fraction of Ki-67^+^ cells in each of these subsets on day 6 of treatment (Fig. 1D). The absolute numbers of CD4^+^ T_reg_ cells were increased by week 3 of IL-15, but this was not statistically significant (Fig. 1E). Thus, combined treatment with IL-15 and CD8^+^ T_CM/E_ clones was safe and increased the circulating endogenous T lymphocytes with a minor transient expansion of CD4^+^ T_reg_ cells.

### Effect of IL-15 on Persistence of Transferred CD8^+^ T_CM/E_ Clones

**Figure 2:**
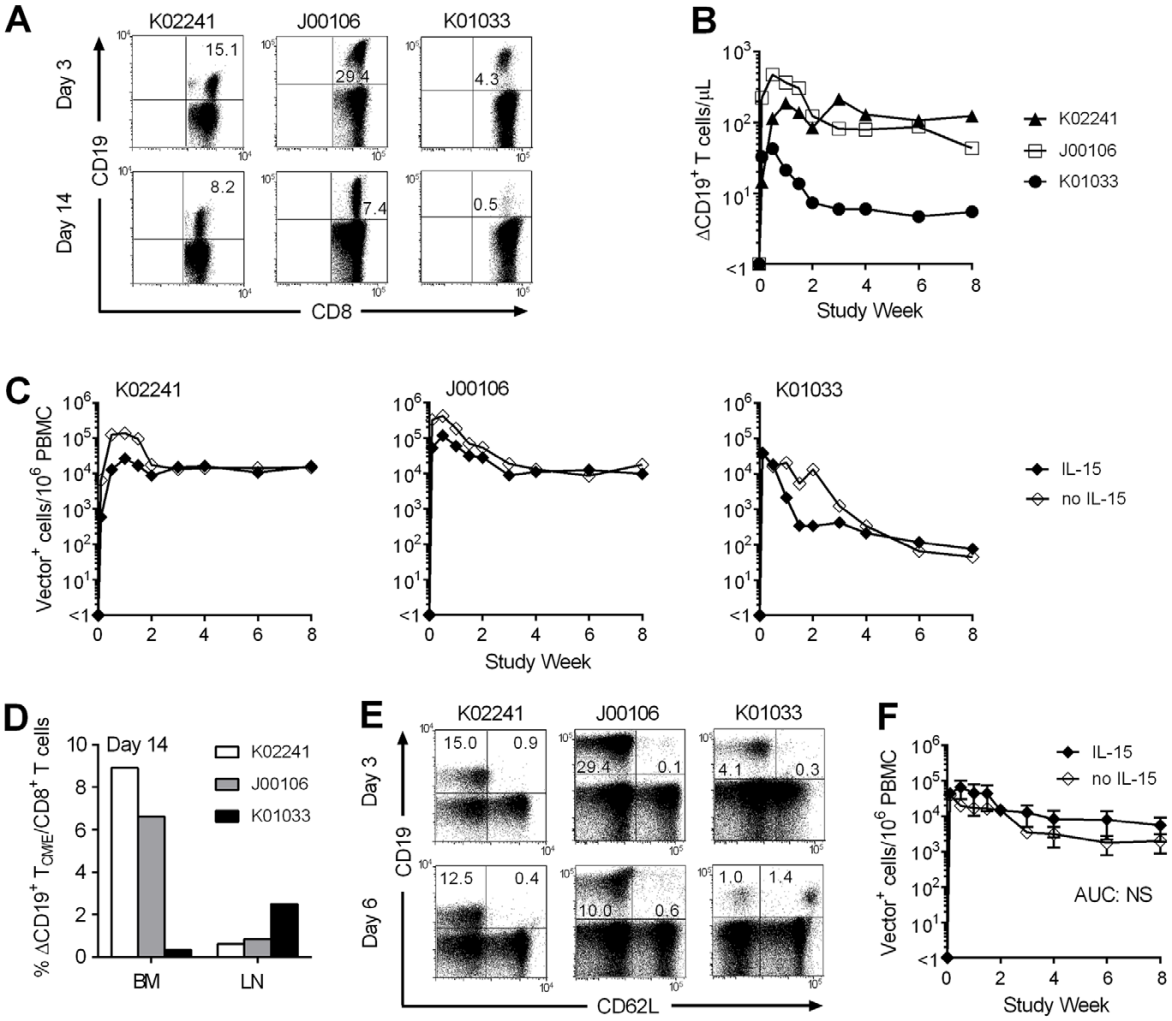
Persistence and migration of CMV-specific CD8^+^ T_CM/E_ clones transferred with or without IL-15. (A,B) Flow cytometric analysis. PBMC were obtained before and after infusion of a ΔCD19^+^CD8^+^ T_CM/E_ clone (5×10^8^/kg) with IL-15. The frequency of transferred ΔCD19^+^CD8^+^ T cells (%) within the CD3^+^CD8^+^ subset was determined by flow cytometry after staining with mAbs specific for CD3, CD8, and CD19. (A) Shown are representative data of PBMC obtained on day 3 and 14. (B) Absolute numbers of persisting ΔCD19^+^ T_CM/E_ cells in the peripheral blood. (C) Real-time qPCR. DNA was isolated from PBMC collected from each macaque before and after the ΔCD19^+^ T_CM/E_ infusion with IL-15. Samples were examined by real-time qPCR for the presence of ΔCD19^+^ T cells (Vector^+^ cells) given with IL-15 (closed diamond). A second infusion of the identical CD20-marked T_CM/E_ clone was given to each of the animals without IL-15 (open diamond). The frequency of CD20^+^ (Vector^+^) T cells in PBMC was assessed by qPCR. Persistence data are presented as overlay. (D) Migration. BM and LNs samples were obtained from each macaque on day 14 after the ΔCD19^+^CD8^+^ T_CM/E_ infusion with IL-15, stained with mAbs specific for CD3, CD8, and CD19, and examined by flow cytometry after gating on CD3^+^CD8^+^ cells. (E) CD62L expression. PBMC obtained on day 3 and 6 after the ΔCD19^+^CD8^+^ T_CME_ transfer with IL-15, were stained with mAbs to detect CD3, CD8, CD19, and CD62L, and examined by flow cytometry after gating on CD3^+^CD8^+^ cells. (F) Persistence of polyclonal CD8^+^ T_CM/E_ cells without or with IL-15 in the peripheral blood. Each of 4 macaques received sequential infusions of either CD20^+^ or ΔCD19^+^ polyclonal CD8^+^ T_CM/E_ cells (5×10^8^/kg) without (open diamond) or with IL-15 (closed diamond). Pre- and post-infusion DNA samples were analyzed by real-time qPCR for the presence of vector^+^ T cells. Shown are mean ± SEM. NS, not significant.

The persistence of transferred ΔCD19-marked T_CM/E_ clones administered with IL-15 in blood, bone marrow (BM), and lymph nodes (LNs) was determined by flow cytometry and qPCR for retroviral vector sequences [13], [27]. On day 3 after the infusion, ΔCD19^+^CD8^+^ T_CM/E_ cells were present in the blood at a mean frequency of 16.3% of CD8^+^ T cells (range, 4.3%−29.4%), corresponding to a mean absolute number of 211 cells/µL (43−476 cells/µL) (Fig. 2A, B). Macaques K02241 and J00106 had the highest peak level of ΔCD19^+^CD8^+^ T cells, which gradually declined to 129 and 79 cells/µL (16,363 and 11,023 vector^+^ cells/10^6^ PBMC) at week four after the T_CM/E_ infusion, and remained at or near this frequency at week eight (Fig. 2B, C). Macaque K01033 had a lower peak frequency of transferred ΔCD19^+^ T_CM/E_ cells, and exhibited a more marked decline to a stable level of 6 cells/µL (211 vector^+^ cells/10^6^ PBMC) at week 4 (Fig. 2B, C).

On day 14 after infusion, the ΔCD19^+^CD8^+^ T_CM/E_ cells were present in the BM and LNs at a mean frequency of 5.3% (range, 0.4%−8.9%) and 1.3% (0.6%−2.5%) of CD8^+^ T cells, respectively (Fig. 2D). Macaque K01033 had the lowest numbers of transferred T cells in the blood and BM, but the highest frequency in the LNs (Fig. 2D). Expression of CD62L on each of the T_CM/E_ clones prior to infusion was minimal (data not shown), however a much higher proportion of ΔCD19^+^ T cells in the blood of K01033 had reacquired CD62L expression on day 6 after transfer compared to the other two animals (Fig. 2E), which may have directed preferential localization of the transferred T_CM/E_ cells to the LNs in this animal. These results show that administering IL-15 with adoptively transferred T_CM/E_ clones did not interfere with establishing a durable antigen-specific T cell response in blood, BM, and LNs, although the frequency of the persisting T cells in each of the sites varied in individual animals.

The mean frequency of the transferred T_CM/E_ clones in the blood at week 4 and 8 in two of the three animals was higher (∼1 log_10_) than that observed in three historical animals that previously received CMV-specific CD8^+^ T_CM/E_ clones without IL-15 [13], suggesting a potential benefit of IL-15 for promoting T cell survival. To specifically address the effect of IL-15 on the survival of transferred T cells, aliquots of the identical CD8^+^ T_CM/E_ clone from each macaque were marked with the CD20 retrovirus and reinfused without IL-15. In contrast to the infusion with IL-15, the absolute numbers and Ki-67 expression of endogenous CD4^+^ and CD8^+^ T cells, NK cells, or CD4^+^ T_reg_ cells were not statistically different after the T_CM/E_ infusion (data not shown). Using qPCR assays for unique ΔCD19 or CD20 vector sequences to analyze cell persistence [27], we observed in all three animals nearly identical levels of transferred CD20^+^ T cells in the blood after the T_CM/E_ infusion without IL-15 compared to the same cell dose of ΔCD19 marked cells administered with IL-15 (Fig. 2C). The CD20^+^ T cells also migrated to BM and LNs at similar levels to that observed after the infusions with IL-15 (data not shown). Thus, the administration of IL-15 in the regimen and dose used here did not appear to substantially influence the persistence of clonally derived CD8^+^ T_CM/E_ cells in the blood or their migration to memory niches.

### Effect of IL-15 on Adoptively Transferred Polyclonal CD8^+^ T_CM/E_ Cells

Most cellular immunotherapy applications are employing polyclonal rather than clonally derived T cells, and it is possible that IL-15 may have more profound effects on the persistence of polyclonal T_CM/E_ cells that are cultured for shorter periods of time ex vivo before transfer. To examine this possibility, we sort-purified polyclonal CD8^+^ T_CM_ cells from four animals and transduced aliquots to express ΔCD19 or CD20 marker genes before infusion. Three animals (A07130, M06259, M07191) were first administered CD20^+^CD8^+^ T_CM/E_ cells (5×10^8^ T cells/kg) alone followed >8 weeks later by a second infusion of the same dose of ΔCD19^+^CD8^+^ T_CM/E_ cells given with a 3-week course of subcutaneous IL-15 [25]. The fourth animal (A09118) received the ΔCD19-marked T_CM/E_ cells without IL-15 first, and then CD20-marked T_CM/E_ cells with IL-15 to control for any potential differences conferred by expression of ΔCD19 or CD20 on T cells, although no effects of these markers have been observed in prior studies [13], [27]. There were no toxicities except a pruritic rash in macaque M06259. A statistically significant increase in absolute numbers and Ki-67 expression of circulating CD8^+^ and CD4^+^ T cells and NK cells was observed during the 3-week IL-15 treatment (data not shown).

One week after the T_CM/E_ infusion, the mean frequency of transferred T cells given without IL-15 was 16,822 vector^+^ cells/10^6^ PBMC and the T cells remained present at a mean frequency of 1,975 vector^+^ cells/10^6^ PBMC at week eight (Fig. 2F). One and eight weeks after the polyclonal T_CM/E_ transfer with IL-15, we detected a mean of 44,531 and 5,557 vector^+^ cells/10^6^ PBMC, respectively (Fig. 2F). Although the mean levels of transferred T cells in the blood were slightly higher when the cells were administered with IL-15, this increment reflected a particularly high level in one of the four animals and the difference in the AUC during the eight-week follow-up was not statistically significant in this sample size (*P* = 0.16). Administration of IL-15 did not augment the magnitude of the polyclonal T_CM/E_ cells previously transferred without IL-15 in these 4 animals (data not shown). Thus, similar to the results obtained with CD8^+^ T_CM/E_ clones, IL-15 had minimal effects on the magnitude or persistence of adoptively transferred polyclonal CD8^+^ T_CM/E_ cells.

### IL-15 Maintains Proliferation of Transferred T Cells, but the Proliferating Fraction Exhibits Enhanced Cell Death

**Figure 3:**
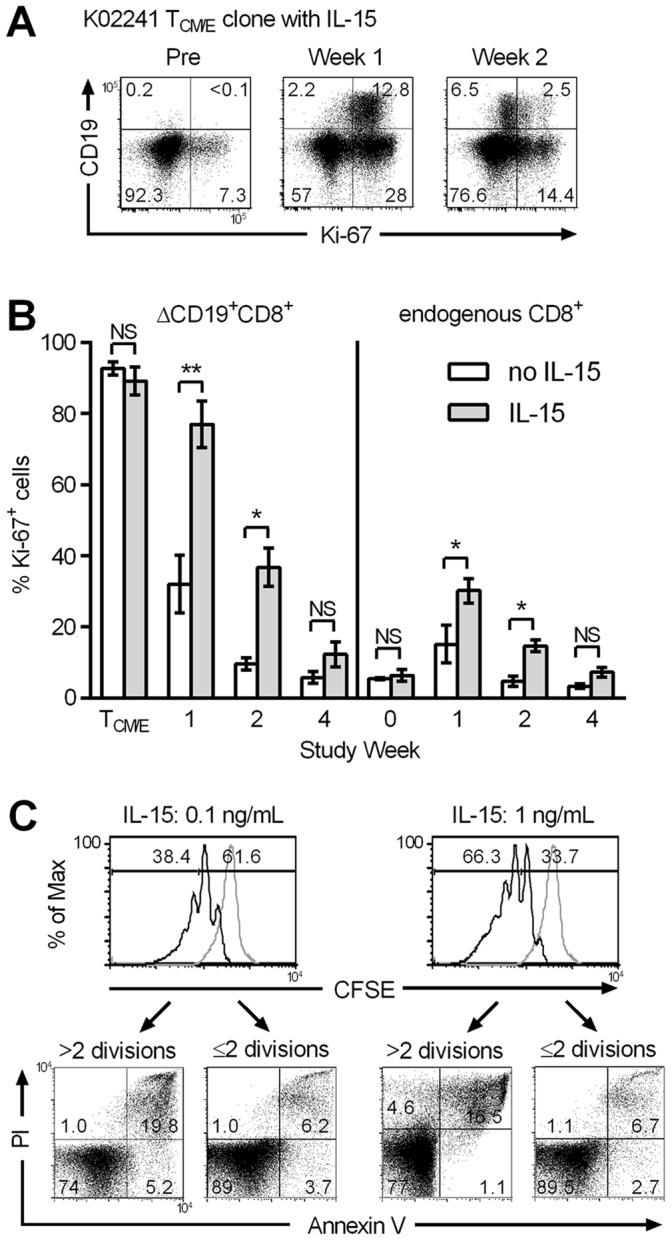
IL-15 maintains transferred T cell proliferation, but promotes in vitro-death of dividing T_CM/E_. (A,B) IL-15 treatment promotes Ki-67 expression of transferred CD8^+^ T_CM/E_ and endogenous T cells. (A) Representative staining of PBMC obtained from macaque K02241 before and at the indicated times after the ΔCD19^+^CD8^+^ T_CM/E_ clone infusion with IL-15. Aliquots of PBMC were examined by flow cytometry after staining for cell-surface expression of CD3, CD8, and CD19, and for intracellular Ki-67 after gating for CD3^+^CD8^+^ T cells. (B) Autologous ΔCD19^+^CD8^+^ T_CM/E_ clones or polyclonal T_CM/E_ cells were infused without IL-15 (4 animals) or with IL-15 (6 animals). Aliquots of PBMC were examined by flow cytometry after staining for cell-surface expression of CD3, CD8, and CD19, and for intracellular Ki-67 after gating for CD3^+^CD8^+^ endogenous (ΔCD19^–^) T cells or transferred ΔCD19^+^ T cells. Data shown are the percentage (%) Ki-67^+^ cells in each subset (mean ± SEM) in animals that did not receive IL-15 (white bars), and those that received IL-15 (gray bars). NS, not significant; *P<0.05; **P<0.01. (C) In vitro proliferation and survival of a representative CMV-specific CD8^+^ T_CM/E_ clone labeled with CFSE, and cultured for 21 days in media alone, or media containing 0.1 or 1 ng/mL of IL-15, respectively. Upper panels: CFSE-dilution indicating the proportion of cells that have undergone ≤2 (CFSE^high^) or >2 divisions (CFSE^low^) in the presence of IL-15 (black line). The gray line shows control T cells cultured in media alone. Lower panels: Binding of Annexin V and staining with PI of CFSE-labeled T cells after 3 weeks of rest. Samples are gated on the CFSE^low^ or CFSE^high^ subset. Similar results were obtained with 5 different T_CM/E_ clones from different macaques.

To determine if IL-15 failed to maintain the proliferation of transferred T_CM/E_ cells in vivo, we examined Ki-67 expression in the infused clonally derived and polyclonal ΔCD19^+^CD8^+^ T_CM/E,_ and in PBMC obtained from 6 animals at intervals after infusion with IL-15. The T_CM/E_ cells were nearly exclusively Ki-67 positive (Ki-67^+^) at the time of infusion (mean: 89.2%), and a mean of 76.9% and 36.8% of the ΔCD19^+^CD8^+^ T_CM/E_ cells remained Ki-67^+^ in vivo one and two weeks after transfer, respectively (Fig. 3A, B). The frequency of Ki-67^+^ cells in endogenous ΔCD19^−^CD8^+^ T cells also increased at weeks one and two of IL-15 (Fig. 3A,B). Surface-expression of the CD20 marker is less stable than ΔCD19 [27], therefore we could not compare Ki-67 expression of the transferred T cells in the same animal without IL-15. Thus, we assayed Ki-67 expression on clonal and polyclonal ΔCD19^+^CD8^+^ T_CM/E_ cells that were previously infused to four animals without IL-15 [13]. We observed a similar frequency of Ki-67^+^ΔCD19^+^ T_CM/E_ cells in the infused product, but the proportion of transferred cells that remained Ki-67^+^ declined significantly more rapidly in the first two weeks after transfer (Fig. 3B).

Given the higher fraction of the transferred T cells that remained Ki-67^+^ when administered with IL-15, it was surprising that the absolute number of T cells was not increased compared to infusions without IL-15. To investigate potential mechanisms to explain this, we first examined the fate of the CD8^+^ T_CM/E_ clones cultured in vitro in IL-15. T_CM/E_ clones were labeled with CFSE and cultured in media alone or with IL-15. A majority (>60%) of the cells plated in low-dose IL-15 (0.1 ng/mL) had undergone ≤2 cell divisions, whereas a larger fraction of T cells (>66%) plated in 1 ng/mL underwent >2 cell divisions (Fig. 3C). Surprisingly, the absolute cell numbers observed when the T cells were cultured with 0.1 and 1 ng/mL of IL-15 did not reflect the expected yield based on the number of divisions observed with CFSE-labeled cells (Fig. 1A, 3C). We co-stained aliquots of CFSE-labeled T cells with Annexin V and PI, and found a much larger fraction of Annexin V^+^ cells in the subset that had undergone >2 divisions compared to those that had undergone ≤2 divisions (Fig. 3C). Increased apoptosis in proliferating T cells was confirmed by assessing caspase activation in this subset (data not shown).

**Figure 4:**
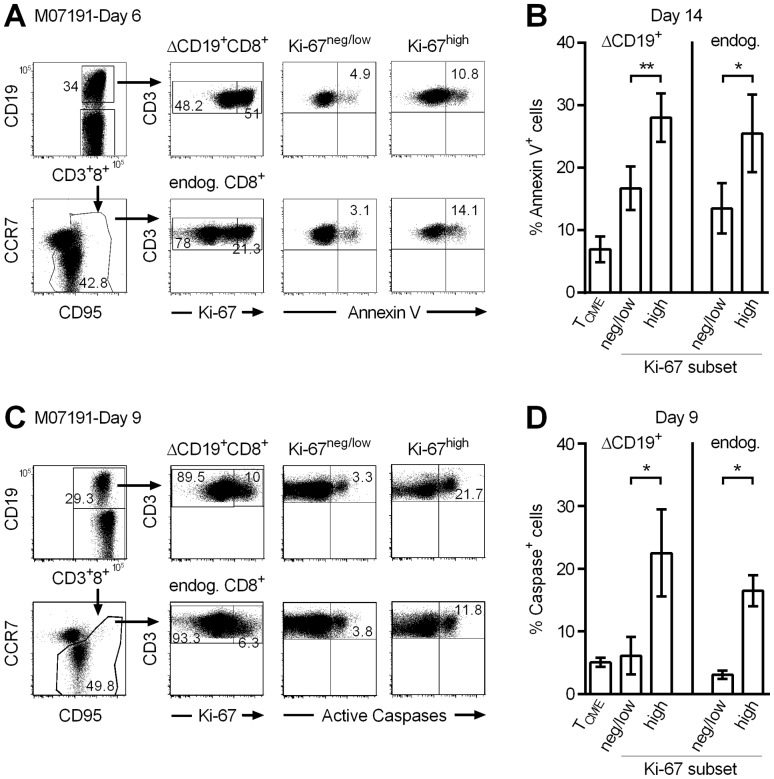
Proliferating Ki-67^high^ **CD8^+^ T_CM/E_ administered with IL-15 display increased signatures of cell death.** (A) PBMC were obtained from M07191 on day 6 after the ΔCD19^+^CD8^+^ T_CM/E_ infusion with IL-15 and stained with mAbs to CD3, CD8, CD19, CCR7 and CD95 to identify transferred ΔCD19^+^CD3^+^CD8^+^ and endogenous ΔCD19^–^CD3^+^CD8^+^ T_M_. Cells were then stained for binding of Annexin V and intracellular Ki-67 and examined by flow cytometry. Inset values show the frequency (%) of T cells in Ki-67^high^ and Ki-67^negative/low^ subsets. Data are gated on CD3^+^CD8^+^ cells. (B) PBMC were obtained 2 weeks after the ΔCD19^+^CD8^+^ T_CM/E_ cell infusion with IL-15 from macaques K02241, J00106, and K01033 and analyzed as described in (A). Shown are mean ± SEM of Annexin V^+^ cells in each subset. *P<0.05; **P<0.01. (C) Representative staining of PBMC for caspase activation. Aliquots of PBMC were obtained on day 9 after the ΔCD19^+^CD8^+^ T_CM/E_ cell infusion with IL-15 (M07191) and assayed for active caspases using a Poly Caspases assay kit. Aliquots were then stained with mAbs to CD3, CD8, CD19, CCR7 and CD95 to identify transferred ΔCD19^+^CD3^+^CD8^+^ and endogenous ΔCD19^–^CD3^+^CD8^+^ T_M_. Cells were also stained for intracellular Ki-67 expression and examined by flow cytometry. Inset values show the frequency (%) of T cells in each subset after gating on transferred ΔCD19^+^CD3^+^CD8^+^ or endogenous ΔCD19^–^CD3^+^CD8^+^ T_M_ cells. (D) PBMC were obtained at the indicated time after the CD8^+^ T_CM/E_ infusion with IL-15 from macaques K02241, J00106, K01033, and M07191, and analyzed as described in (C). Shown are mean ± SEM of active caspases^+^ cells (%) in each subset. *P<0.05.

The in vitro findings suggested that the cell division promoted by IL-15 was balanced by cell death. However, detecting apoptotic cells in vivo is challenging because apoptotic cells are targeted for rapid clearance [39]. Exposure of phosphatidyl-serine on the exterior cell membrane is an early event in apoptosis and can be detected by cell-surface binding of Annexin V [40]. Thus, we examined if the transferred CD8^+^ T_CM/E_ cells that maintained Ki-67 expression in vivo in response to IL-15, preferentially bound Annexin V. Samples of PBMC from macaque K02241, J00106, K01033, and M07191 were obtained 1–2 weeks after the ΔCD19^+^ T_CM/E_ infusion with IL-15, and stained with mAbs to identify ΔCD19^+^CD3^+^CD8^+^ and endogenous ΔCD19^−^CD3^+^CD8^+^ T_M_ cells, and for binding of Annexin V and intracellular Ki-67 expression (Fig. 4A, B). We found a significantly increased frequency of Annexin V^+^ cells in the Ki-67^high^ subset compared to the Ki-67^negative/low^ fraction in the endogenous ΔCD19^−^CD8^+^ T_M_ cell subset containing both antigen-experienced T_CM_ and T_EM,_ and the transferred ΔCD19^+^CD8^+^ T_CM/E_ subset (Fig. 4A, B). Consistent with this, the analysis of samples of post-infusion PBMC demonstrated a significantly increased frequency of T cells with activated caspases in the subset of proliferating Ki-67^ high^ endogenous ΔCD19^−^CD8^+^ T_M_ and transferred ΔCD19^+^CD8^+^ T cells (Fig. 4C, D). We also performed a precise subset analysis of the level of apoptosis in endogenous CD8^+^CCR7^+^CD95^+^ central memory and CD8^+^CCR7^−^CD95^+^ effector memory T cells and found an increased level of apoptosis was detectable in the Ki-67^ high^ fraction of both memory subsets (Fig. S3A–D). Collectively, the results demonstrate that adoptively transferred T cells induced to proliferate in vivo in response to IL-15 undergo a compensatory activation of apoptosis, presumably as a homeostatic mechanism to maintain T cell numbers.

### IL-15 does not Interfere with Establishing Long-lived T_CM_ and T_EM_ Cells by Transfer of Antigen-specific T_CM/E_ Clones

**Figure 5:**
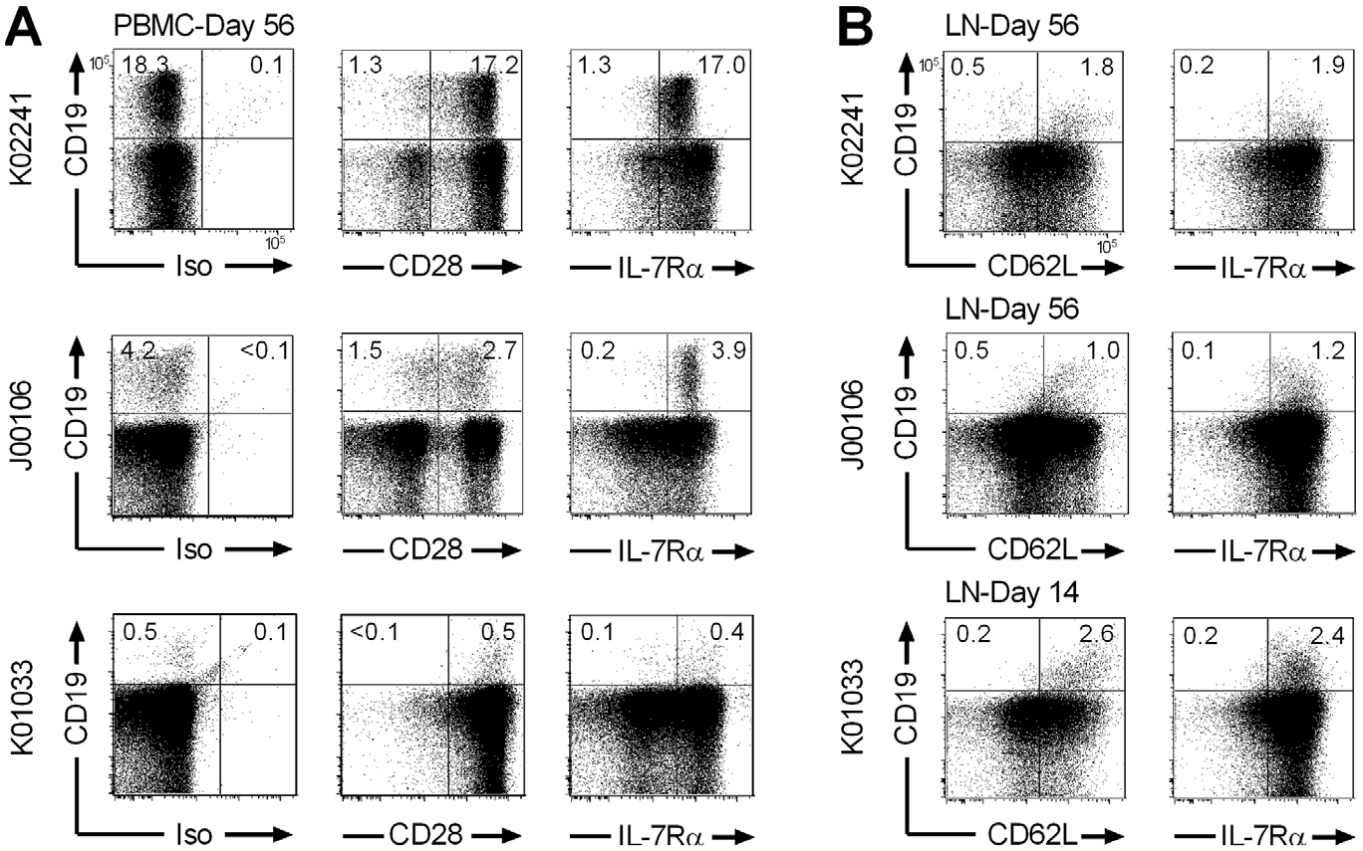
CD8^+^ T_CM/E_ clones transferred with IL-15 acquire memory-marker and migrate to T_M_ niches. (A, B) Samples of PBMC (A) or LNs (B) were obtained from macaques K02241, J00106, or K01033 at the indicated days after the infusion of a ΔCD19^+^CD8^+^ T_CM/E_ clone with IL-15 and stained with mAbs specific for CD3, CD8, and CD19, and for CD28, IL-7Rα, and/or CD62L respectively. Inset values show the frequency (%) of T cells in each subset after gating on CD3^+^CD8^+^ cells.

We next examined whether the balanced proliferation and cell death induced by IL-15 altered the ability of the antigen-specific CD8^+^ T_CM/E_ clones to establish long-term T cell memory and persist long-term. In prior studies, we showed that antigen-specific CD8^+^ T_CM/E_ clones transferred without IL-15 reacquired CD28 and CD127 (IL-7Rα) expression, and established reservoirs of CD62L^+^ T_M_ in LNs [13]. In each of the three animals treated with ΔCD19^+^CD8^+^ T_CM/E_ clones with IL-15, a majority of the persisting T cells reacquired a CD28^+^IL-7Rα^+^ phenotype (Fig. 5A), and notable populations of CD62L^+^IL-7Rα^+^ΔCD19^+^ T cells were present in LNs obtained 14–56 days post-infusion in all three animals (Fig. 5B). Transferred polyclonal ΔCD19^+^CD8^+^ T_CM/E_ cells also migrated to BM and LNs regardless of the cytokine treatment and acquired memory markers (Fig. S4).

**Figure 6:**
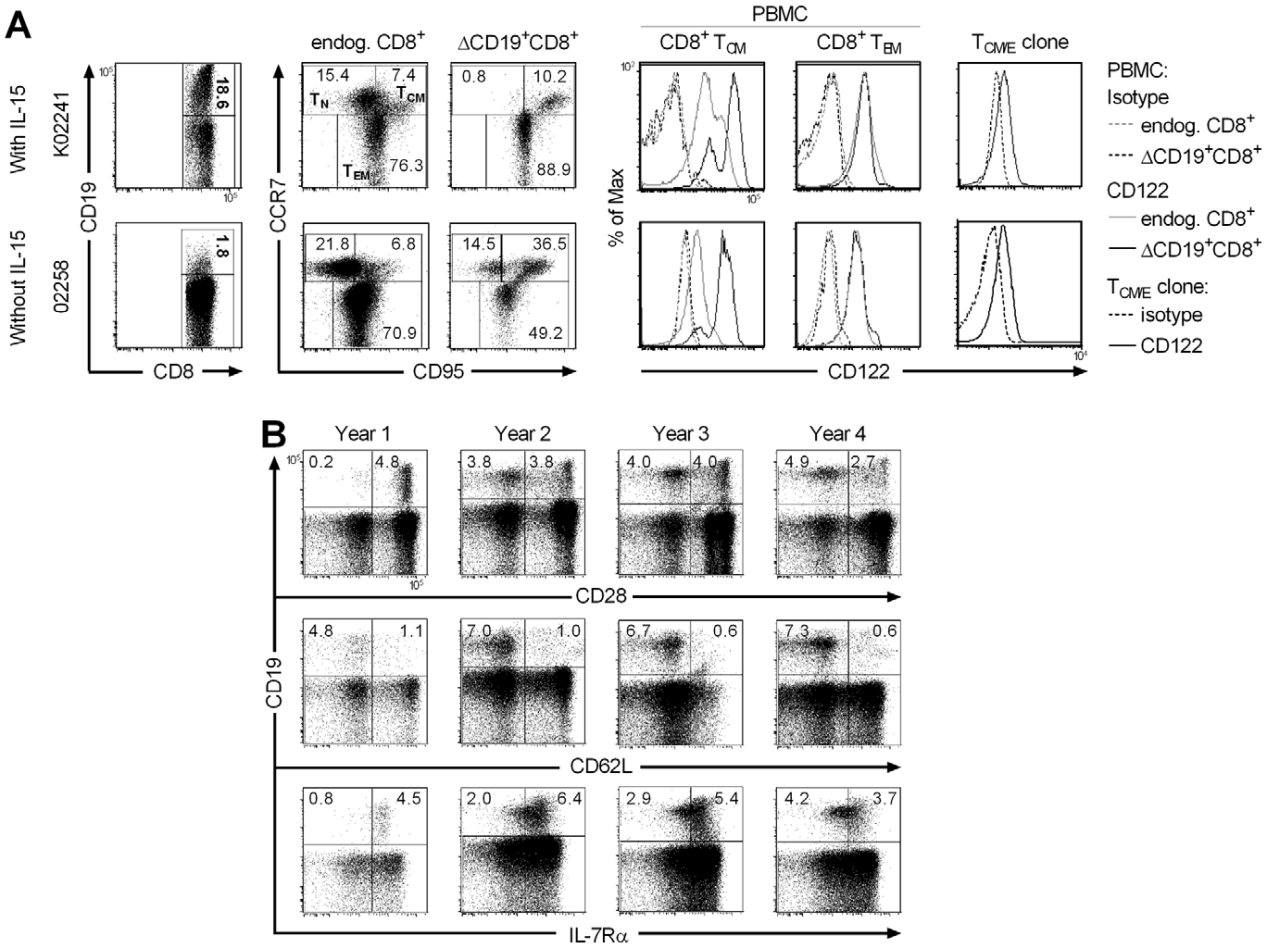
T_CM/E_ adopt CD122^intermediate^ T_EM_, and CD122^intermediate/high^ T_CM_-phenotypes regardless of IL-15 and persist long-term. (A) Samples of PBMC were obtained >8 weeks after adoptive transfer of a ΔCD19^+^CD8^+^ T_CM/E_ clone with or without IL-15. ΔCD19^+^CD8^+^ T_CM/E_ were identified in PBMC by staining with mAbs to CD3, CD8, and CD19, and anti-CCR7 and anti-CD95 mAbs were used to identify CCR7^+^CD95^+^ T_CM_ and CCR7^–^CD95^+^ T_EM_ subsets. Inset values show the frequency (%) of T cells in each subset. Cells were also co-stained with an anti-CD122 mAb to determine the expression of CD122 in the endogenous ΔCD19^–^CD3^+^CD8^+^ (gray solid line) and transferred ΔCD19^+^CD3^+^CD8^+^ (black solid line) T_M_ subsets. Isotype-matched mAbs were used as control (black/gray dashed lines). Expression of CD122 (back solid line) and the isotype control (black dashed line) on the infused T_CM/E_ clone is shown in the far right panels. Data are representative for results in 5 animals receiving infusions of ΔCD19^+^CD8^+^ T_CM/E_ cells with or without IL-15. (B) PBMC were obtained from macaque K02241 at yearly intervals after the infusion of a ΔCD19^+^CD8^+^ T_CM/E_ clone with IL-15. The frequency of the transferred ΔCD19^+^ T cells (%) within the CD3^+^CD8^+^ T cell subset was determined by flow cytometry after staining with mAbs to detect expression of CD3, CD8, and CD19. Samples were co-stained using mAbs to detect expression of CD28, CD62L, and IL-7Rα, and examined by flow cytometry after gating on CD3^+^CD8^+^ T cells. Inset values show representative data of the frequency (%) of T cells in each subset.

The expression of high levels of CD122 on a subset of T_M_ cells has recently been suggested, among other markers, to identify a rare “memory stem cell” (T_SCM_) subset in mice and humans [41], [42]. In macaques, there is also a minor fraction of CD122^high^ cells in the endogenous CD8^+^CCR7^+^CD95^+^ T_CM_ subset (Fig. 6A). Retrospectively, we could not determine whether the transferred T_CM/E_ clones that persisted long-term in vivo were derived from a CD122^high^ T cell since this was not one of the selection parameters. However, both in animals that received IL-15 and those that did not, a large fraction of ΔCD19^+^ T_CM/E_ cells that adopted a CCR7^+^ T_CM_ phenotype in vivo and persisted >8 weeks, exhibited a CD122^high^ phenotype (Fig. 6A). The level of CD122-expression on transferred T cells that remained CCR7^–^ was nearly identical to the endogenous CCR7^–^ T_EM_ (Fig. 6A). Thus, the transfer of T_E_ cells derived from a single CD8^+^ T_CM_ cell repopulates both CD122^high^ and CD122^intermediate^ T_CM_ and T_EM_ subsets, irrespective of IL-15 administration after the T cell infusion.

The longevity of CD8^+^ T_CM/E_ clones given with IL-15 was examined in two animals (K02241, J00106) that were followed for >4 years after the T_CM/E_ infusion. We observed a stable level of ΔCD19^+^ T cells in the blood that were predominantly IL-7Rα^+^, and contained CD62L^+^, CCR7^+^, and CD28^+^ subsets (Fig. 6B and data not shown). Because the T_CM/E_ clones were transduced with the ΔCD19 retrovirus during expansion, the infused cell product contained multiple distinct retroviral insertion sites. To exclude the possibility that long-term persistence reflected the selective outgrowth of one or a few clonal progeny driven by IL-15 or by a mutation induced by retrovirus insertion, we mapped the transgene insertion sites in both the infused and persisting cells present in these two animals (K02241 and J00106) 10−12 months after infusion of the ΔCD19^+^CD8^+^ T_CM/E_ clones. Multiple insertion sites were present in the persisting ΔCD19^+^ T cells in both of the animals, demonstrating that the surviving T cells represented diverse progeny of a single T_CM_ cell and excluding selective expansion of one or a few transduced T cells (Table 1).

**Table 1:** Analysis of unique RIS in adoptively transferred and persisting CMV-specific ΔCD19^+^CD8^+^ T_CM/E_ clones.

Macaque	Infused ΔCD19^+^ T_CM/E_ Cells			Post-infusion PBMC (10–12 months)		
	Unique RIS/Reads^1^	Range Top 1–10 (%)^2^	Single Capture (%)^3^	Unique RIS/Reads	Range Top 1–10 (%)	Single Capture (%)
K02241	4,751/21,571	0.27–0.17	32.7	823/48,550	2.12–1.27	23.7
J00106	863/1,400	0.79–0.43	65.9	683/1,752	2.45–0.63	46.6

1 **Unique RIS/Reads:** Number of unique RIS over the number of total “qualified” sequence reads.

2 **Range Top 1–10 (%):** The percentage of the total “qualified” sequence reads of the most frequently sequenced RIS “Top 1” to the 10^th^ most frequently sequenced RIS “Top 10”.

3 **Single Capture (%):** The percentage of the “Unique RIS” that were sequenced only one time. A lower number corresponds to more complete coverage of the Unique RIS in that given sample.

## Discussion

Our study compared the transfer of defined CD8^+^ T_CM/E_ populations with and without IL-15 in seven macaques, and provided an internal control for any effects of IL-15 on the adoptively transferred CD8^+^ T cells. The results show that T cell transfer combined with a 3-week regimen of subcutaneous IL-15 in lymphoreplete macaques was safe, yielded plasma IL-15 levels above that necessary for maintaining CD8^+^ T_CM/E_ cell survival in vitro, and induced or maintained proliferation of endogenous and transferred lymphocytes in vivo. IL-15 did not interfere with the persistence of the transferred CD8^+^ T_CM/E_ cells and the capacity to reacquire phenotypic markers of T_M_ cells, but failed to augment the frequency of the transferred CD8^+^ T_CM/E_ in the circulation at early and late times after T cell transfer. Due to a limited number of animals and potential variability between cell infusions, it was not possible to provide precise quantitation of the magnitude of the transferred long-term memory response that persisted prolonged after infusions without or with IL-15.

The observation that IL-15 promoted the proliferation of the transferred T cells but had no major effect on the magnitude of the transferred T cell persistence in blood, BM, or LNs was unexpected. Prior studies in murine models examined the effects of IL-15 either supplemented in vitro, by exogenous administration, or constitutively expressed by the transferred T cells, and suggested a beneficial effect on the transferred T cell function and/or survival [14], [43]–[47]. For example, in the pmel-1 T cell receptor transgenic mouse model of adoptive T cell transfer, the exogenous administration of both IL-15 and IL-2 improved the anti-tumor response during the 34 days of follow-up [43]. The addition of IL-15 to the culture media in vitro during T cell activation or its expression as a transgene in transferred T cells further enhanced the anti-tumor function of the adoptively transferred T cells. Similarly, mice bearing P1A^+^ tumors treated with TCR-transgenic T cells and low-dose IL-15 had longer persistence of tumor-specific CD8^+^ T cells compared to controls [44]. Teague et al. showed that culturing tolerant tumor-specific CD8^+^ T cells in vitro in IL-15 induces proliferation and reverses tolerance enabling effective tumor therapy after adoptive transfer, but gave IL-2 to support T cells in vivo [45]. There are notable differences between these murine tumor models and the macaque model that we used. The murine models examined settings in which the T cells would encounter antigen in vivo immediately after adoptive transfer with IL-15, whereas our studies examined how IL-15 altered the fate of transferred T cells that may not immediately encounter antigen. Thus, IL-15 may be useful to augment an immune response where antigen is abundant, but our data would suggest that at the doses used here IL-15 does not provide a major benefit in settings where antigen is limited.

It is possible that the inability of IL-15 to increase T cell persistence in lymphoreplete macaques could reflect migration of the infused T_CM/E_ cells to tissue sites, increased cell death, or both [34], [35]. We did not observe a decline in endogenous or transferred lymphocyte numbers during treatment with IL-15, making selective tissue emigration of the transferred CD8^+^ T_CM/E_ cells unlikely, and we found a similar frequency of transferred T_CM/E_ in BM and LNs. However, a higher fraction of the T_CM/E_ cells, which proliferated in response to IL-15 in vitro and in vivo, expressed Annexin V and activated caspases, indicating these cells were destined to undergo apoptosis. Studies in murine models identified proliferation-linked apoptosis as a mechanism for regulating CD8^+^ T cell homeostasis and maintaining a quantitatively stable memory pool in normal hosts [48]. Similarly, in rhesus macaques treated with daily IL-15, Ki-67^+^ endogenous lymphocytes displayed increased Annexin V binding [35]. Thus, increased apoptosis of both transferred and endogenous T cells driven to proliferate by IL-15 may represent a compensatory mechanism to limit T cell expansion and maintain T cell homeostasis in lymphoreplete hosts. It is possible that higher or sustained steady-state doses of IL-15 alone or combined with lymphodepletion or antigen-stimulation, or local delivery of the cytokine, would enhance the systemic or local frequency of transferred antigen-specific T cells at diseased tissues as suggested by murine studies.

Our study of the persistence and migration of transferred T_CM/E_ cells alone and with IL-15 was facilitated by retroviral gene marking, which provided a non-immunogenic cell-surface marker for tracking cells in vivo by flow cytometry and unique sequences for qPCR and provided the opportunity to examine the progeny of a single cell long-term in vivo [13], [27]. In animals that received CD8^+^ T_CM/E_ clones transduced during clonal expansion, we mapped the RIS to examine the diversity of T cells that persisted in vivo. The results demonstrate that the progeny of a single T_CM_ cell forms both T_CM_ and T_EM_ subsets and persists for several years in vivo. Importantly, we observed that diversity in RIS was maintained and no evidence of oligoclonal expansions or vector-induced transformation.

The factors that govern the fate of the ex vivo isolated and expanded T_E_ cells after infusion in outbred animals or humans have not been fully elucidated. Our previous studies illustrated the importance of cell-intrinsic factors for determining survival of antigen-specific CD8^+^ T_E_ clones from different T_M_ subsets [13]. Even when T_CM_ are used to derive T_E_ cells, >1–2 log_10_ differences in the level of T cells in the circulation were observed after the infusion of individual CD8^+^ T_CM/E_ clones or lines in lymphoreplete animals. Here, we show that the level of T cells in the blood achieved by cell transfer is reproducible with repeated infusions and not affected by IL-15 administration, suggesting that cell-intrinsic or host-intrinsic factors govern the fate of adoptively transferred T cells in vivo. Studies in mice and with human T cells have suggested a rare CD8^+^CD122^high^ T_SCM_ cell subset is uniquely capable of restoring high levels of T_CM_, T_EM_, and T_E_ cells in vivo [41], [42]. We found that a subset of the CD8^+^ T_CM/E_ clones that persisted as long-lived memory cells adopted CD122^intermediate^ and CD122^high^ fates regardless of the cytokine treatment. The signals that determine the individual cell phenotypes that develop from infusing the progeny of a single T cell, and the potential conversion between phenotypes are not addressed by our current studies. The NHP model may be suitable for examining these issues in future studies, and provide insights for selecting T cells for adoptive therapy or predicting the behavior of the transferred T cells.

## Supporting Information

Figure S1 **Phenotype of endogenous CD8^+^CD62L^+^ T cells.** PBMC were obtained from macaques K02241, J00106, and K01033, and two control animals M03096, and 02269, respectively, and enriched for CD62L-expression as described in the Method section. Aliquots of the cells were stained with fluorochrome-conjugated anti-CD8, CD3, CD62L, and CCR7 mAbs, and analyzed by flow cytometry after gating on CD3^+^CD8^+^ T cells.
(TIF)

Figure S2 **Characterization of CMV-specific CD8^+^ T_CM/E_ clones.** (A) In vitro growth. CMV-specific CD8^+^ T_CM/E_ clones from macaque K02241 (closed triangle), J00106 (open square), and K01033 (closed circle) used for adoptive transfer were stimulated with anti-CD3/CD28 mAbs, γ-irradiated feeder cells, and IL-2 (50 U/mL). Cell growth was measured by counting viable cells on indicated days. (B) Telomere length. The median telomere length+SD (in kb) of the infused T_CM/E_ clones from each of the macaques was measured by automated flow-FISH. (C) Cytotoxic activity of T_CM/E_ clones was examined in a chromium release assay at effector-to-target ratios of 20∶1(black bars), 10∶1 (hatched bars), or 5∶1 (gray bars) using autologous CMV peptide-pulsed target cells or unpulsed controls (white bars). Peptide sequences were ATTRSLEYK (K02241), NPTDRPIPT (J00106), and DQVRVLILY (K01033).
(TIF)

Figure S3 **Proliferating endogenous CD8^+^ T_CM_ and T_EM_ display increased signatures of cell death during IL-15.** (A) PBMC were obtained from M07191 on day 6 after the ΔCD19^+^CD8^+^ T_CM/E_ infusion with IL-15 and stained with mAbs to CD3, CD8, CD19, CCR7 and CD95 to identify the endogenous ΔCD19^–^CD3^+^CD8^+^ T_M_. Cells were then stained for binding of Annexin V and intracellular Ki-67 and examined by flow cytometry. Inset values show the frequency (%) of T cells in Ki-67^high^ and Ki-67^negative/low^ subsets. Data are gated to identify CCR7^+^CD95^+^ T_CM_ or CCR7^–^CD95^+^ T_EM_ in the endogenous ΔCD19^–^CD3^+^CD8^+^ T cell subset. (B) PBMC were obtained at the indicated time after the ΔCD19^+^CD8^+^ T_CM/E_ infusion with IL-15 from macaques K02241, J00106, and K01033 and analyzed as described in (A). Shown are mean ± SEM of Annexin V^+^ cells in each subset. *P<0.05. (C) Representative staining of PBMC for caspase activation. Aliquots of PBMC were obtained on day 9 after the ΔCD19^+^CD8^+^ T_CM/E_ infusion with IL-15 (M07191) and assayed for active caspases using a Poly Caspases assay kit. Aliquots were then stained with mAbs to CD3, CD8, CD19, CCR7 and CD95 to identify the endogenous ΔCD19^–^CD3^+^CD8^+^ T_M_. Cells were also stained for intracellular Ki-67 expression and examined by flow cytometry. Inset values show the frequency (%) of T cells in each subset after gating on endogenous CCR7^+^CD95^+^ T_CM_ or CCR7^–^CD95^+^ T_EM_ in the ΔCD19^–^CD3^+^CD8^+^ T cell subset. (D) Aliquots of PBMC were obtained from macaques K02241, J00106, K01033, and M07191 at the indicated time after the ΔCD19^+^CD8^+^ T_CM/E_ infusion with IL-15 and assayed for active caspases using a Poly Caspases assay kit. Aliquots were then stained and analyzed as described in (C). Shown are mean ± SEM of active caspases^+^ cells (%) in each subset. *P<0.05; **P<0.01.
(TIF)

Figure S4 **Polyclonal CD8^+^ T_CM/E_ transferred with IL-15 acquire memory-marker and migrate to T_M_ niches.** Samples of PBMC (A), BM (B), or LNs (C) were obtained on day 14 after the infusion of polyclonal ΔCD19^+^CD8^+^ T_CM/E_ cells (5×10^8^/kg) given without IL-15 (A09118) or with intermittent IL-15 administration (A07130). Aliquots were stained with mAbs to detect expression of CD3, CD8, and CD19 and markers expressed by T_M_ cells including CD28, CD62L, and IL-7Rα (CD127), and examined by flow cytometry. Inset values show representative data of the frequency (%) of T cells after gating on CD3^+^CD8^+^ T cells.
(TIF)
