# Supplementary figures and images for: Proliferation-Linked Apoptosis of Adoptively Transferred T Cells after IL-15 Administration in Macaques
Source: PLoS One. 2013 Feb 13;8(2):e56268. doi: 10.1371/journal.pone.0056268 (PMC3572023; doi:10.1371/journal.pone.0056268)

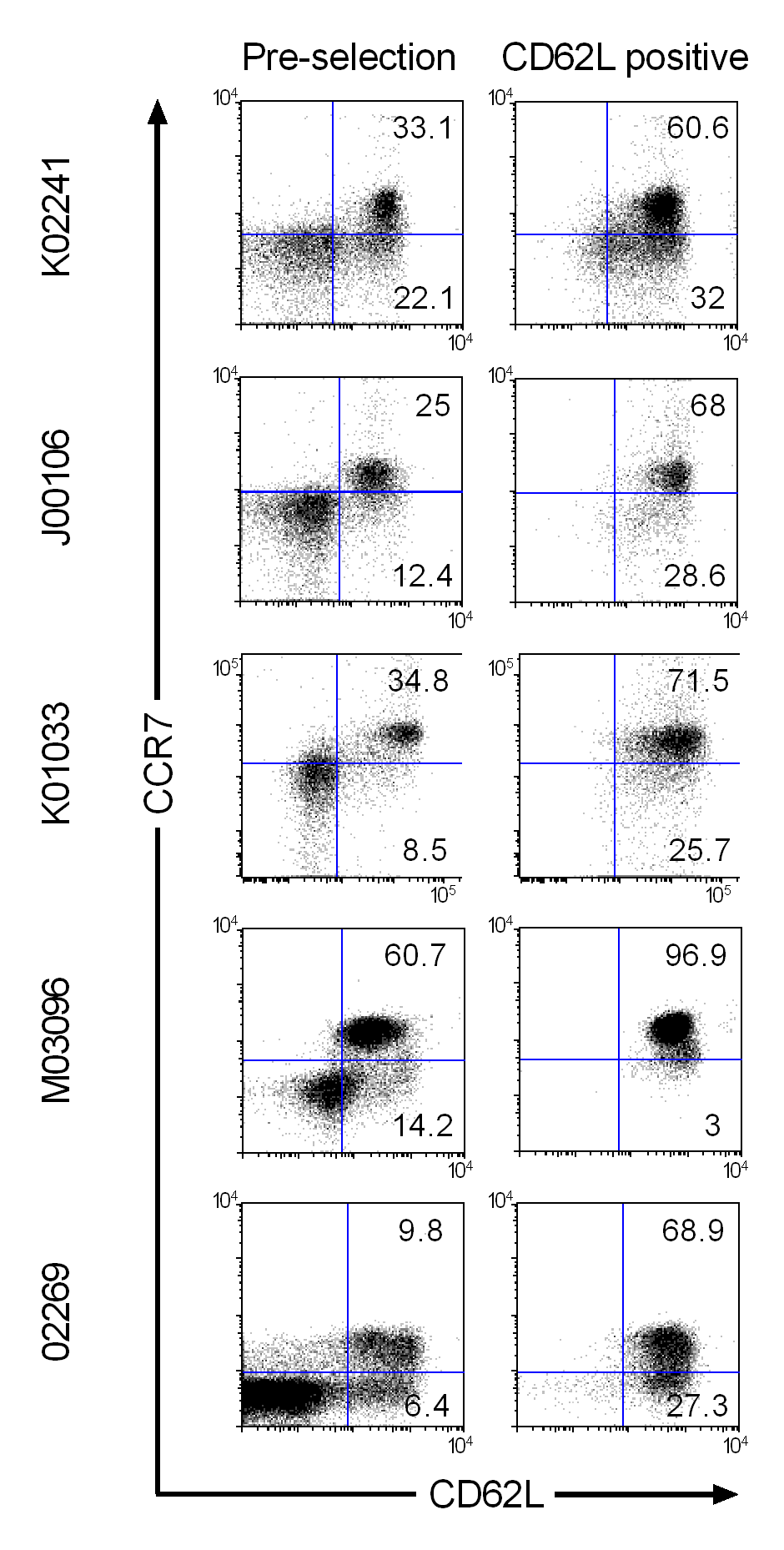

Supplement: Figure S1 — Phenotype of endogenous CD8+CD62L+ T cells. PBMC were obtained from macaques K02241, J00106, and K01033, and two control animals M03096, and 02269, respectively, and enriched for CD62L-expression as described in the Method section. Aliquots of the cells were stained with fluorochrome-conjugated anti-CD8, CD3, CD62L, and CCR7 mAbs, and analyzed by flow cytometry after gating on CD3+CD8+ T cells. (TIF) [file pone.0056268.s001.tif]

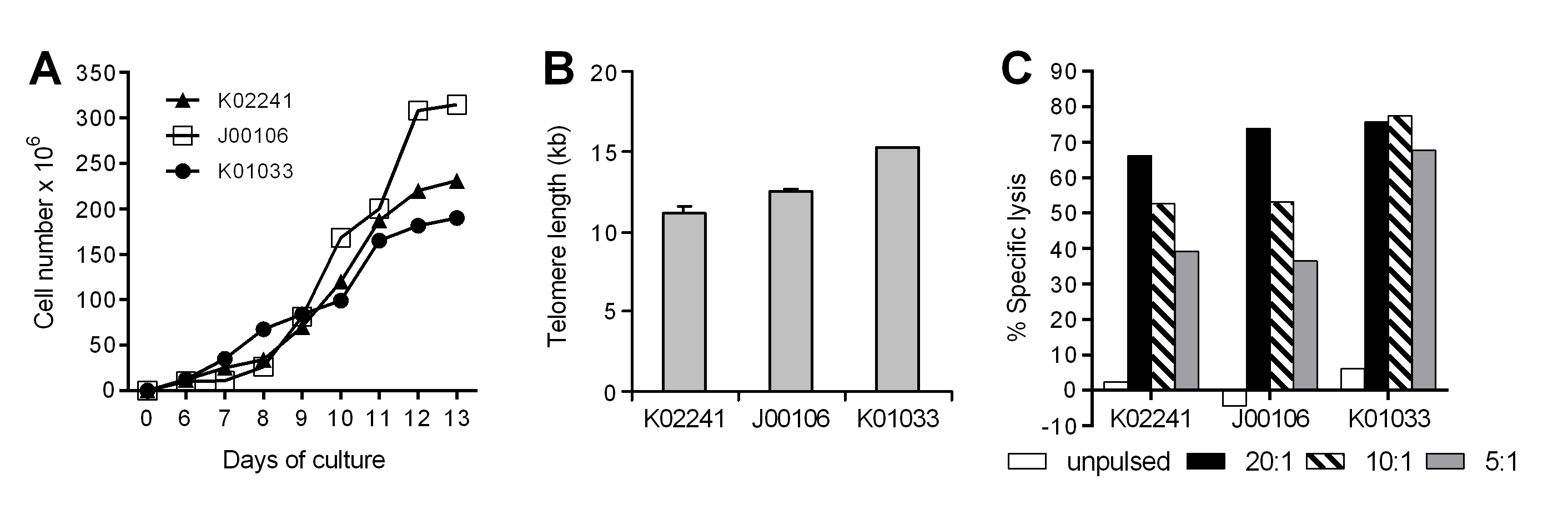

Supplement: Figure S2 — Characterization of CMV-specific CD8+ TCM/E clones. (A) In vitro growth. CMV-specific CD8+ TCM/E clones from macaque K02241 (closed triangle), J00106 (open square), and K01033 (closed circle) used for adoptive transfer were stimulated with anti-CD3/CD28 mAbs, γ-irradiated feeder cells, and IL-2 (50 U/mL). Cell growth was measured by counting viable cells on indicated days. (B) Telomere length. The median telomere length+SD (in kb) of the infused TCM/E clones from each of the macaques was measured by automated flow-FISH. (C) Cytotoxic activity of TCM/E clones was examined in a chromium release assay at effector-to-target ratios of 20∶1(black bars), 10∶1 (hatched bars), or 5∶1 (gray bars) using autologous CMV peptide-pulsed target cells or unpulsed controls (white bars). Peptide sequences were ATTRSLEYK (K02241), NPTDRPIPT (J00106), and DQVRVLILY (K01033). (TIF) [file pone.0056268.s002.tif]

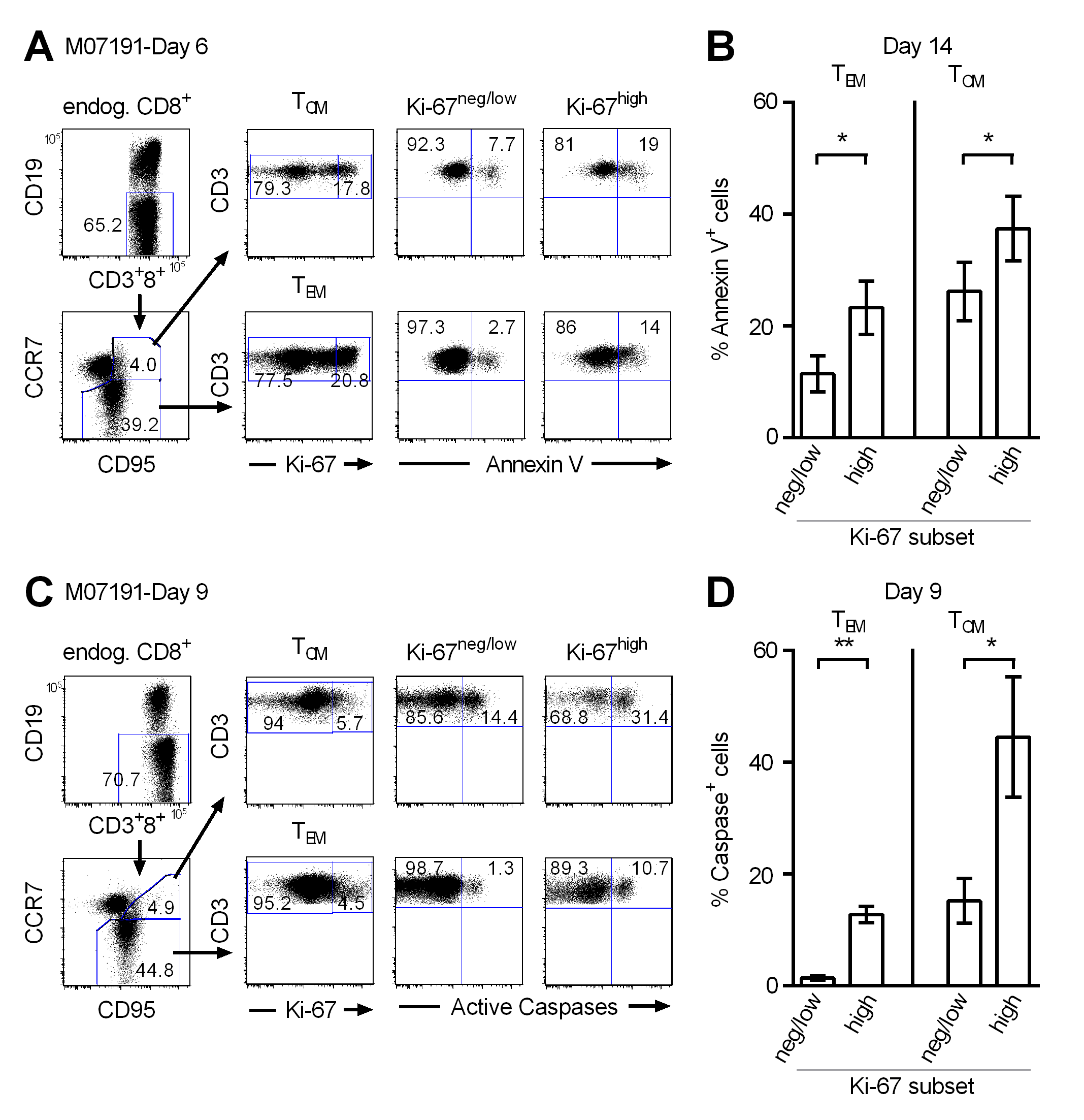

Supplement: Figure S3 — Proliferating endogenous CD8+ TCM and TEM display increased signatures of cell death during IL-15. (A) PBMC were obtained from M07191 on day 6 after the ΔCD19+CD8+ TCM/E infusion with IL-15 and stained with mAbs to CD3, CD8, CD19, CCR7 and CD95 to identify the endogenous ΔCD19–CD3+CD8+ TM. Cells were then stained for binding of Annexin V and intracellular Ki-67 and examined by flow cytometry. Inset values show the frequency (%) of T cells in Ki-67high and Ki-67negative/low subsets. Data are gated to identify CCR7+CD95+ TCM or CCR7–CD95+ TEM in the endogenous ΔCD19–CD3+CD8+ T cell subset. (B) PBMC were obtained at the indicated time after the ΔCD19+CD8+ TCM/E infusion with IL-15 from macaques K02241, J00106, and K01033 and analyzed as described in (A). Shown are mean ± SEM of Annexin V+ cells in each subset. *P<0.05. (C) Representative staining of PBMC for caspase activation. Aliquots of PBMC were obtained on day 9 after the ΔCD19+CD8+ TCM/E infusion with IL-15 (M07191) and assayed for active caspases using a Poly Caspases assay kit. Aliquots were then stained with mAbs to CD3, CD8, CD19, CCR7 and CD95 to identify the endogenous ΔCD19–CD3+CD8+ TM. Cells were also stained for intracellular Ki-67 expression and examined by flow cytometry. Inset values show the frequency (%) of T cells in each subset after gating on endogenous CCR7+CD95+ TCM or CCR7–CD95+ TEM in the ΔCD19–CD3+CD8+ T cell subset. (D) Aliquots of PBMC were obtained from macaques K02241, J00106, K01033, and M07191 at the indicated time after the ΔCD19+CD8+ TCM/E infusion with IL-15 and assayed for active caspases using a Poly Caspases assay kit. Aliquots were then stained and analyzed as described in (C). Shown are mean ± SEM of active caspases+ cells (%) in each subset. *P<0.05; **P<0.01. (TIF) [file pone.0056268.s003.tif]

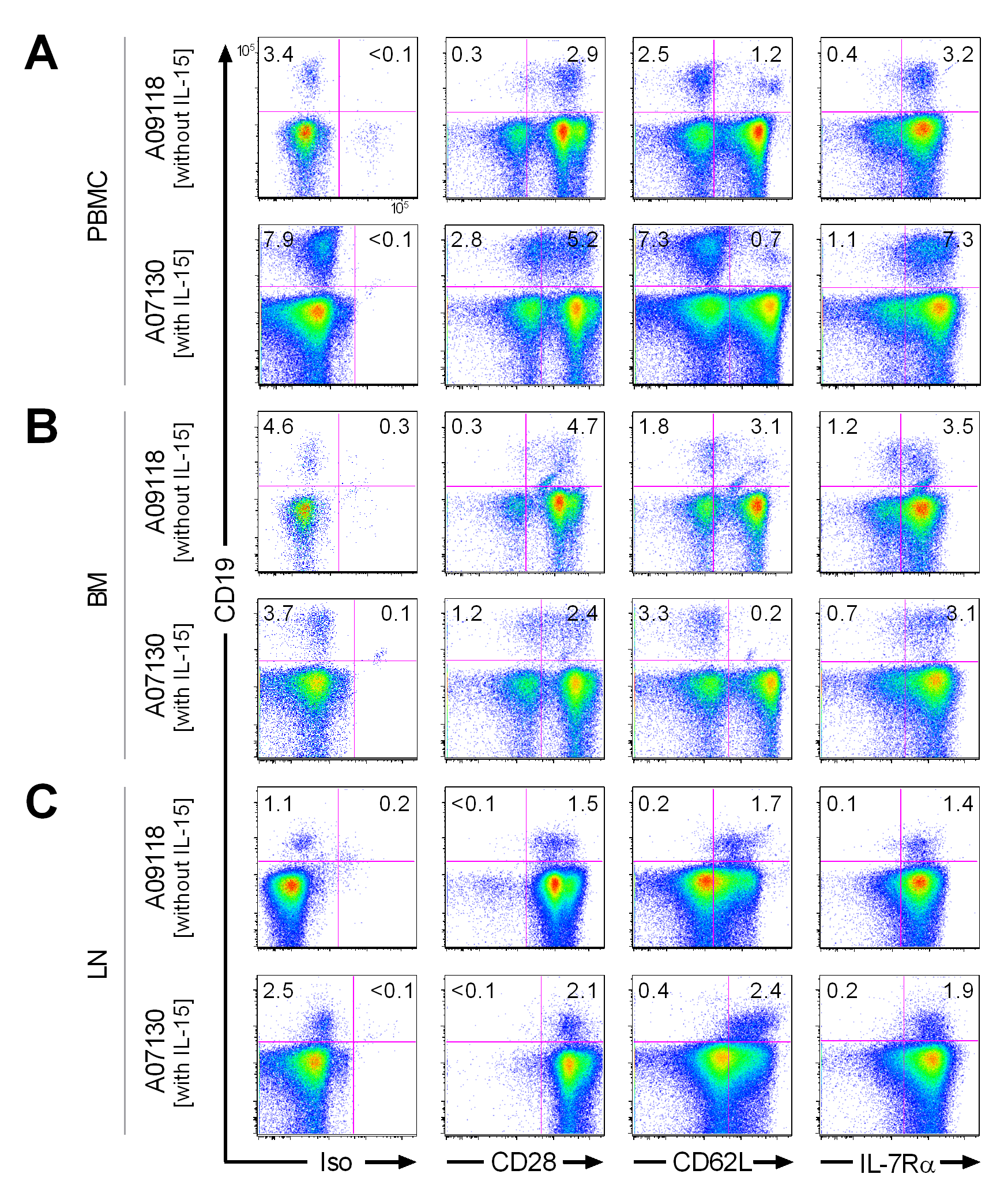

Supplement: Figure S4 — Polyclonal CD8+ TCM/E transferred with IL-15 acquire memory-marker and migrate to TM niches. Samples of PBMC (A), BM (B), or LNs (C) were obtained on day 14 after the infusion of polyclonal ΔCD19+CD8+ TCM/E cells (5×10^8^/kg) given without IL-15 (A09118) or with intermittent IL-15 administration (A07130). Aliquots were stained with mAbs to detect expression of CD3, CD8, and CD19 and markers expressed by TM cells including CD28, CD62L, and IL-7Rα (CD127), and examined by flow cytometry. Inset values show representative data of the frequency (%) of T cells after gating on CD3+CD8+ T cells. (TIF) [file pone.0056268.s004.tif]
